# Supplementary material for: Single reconstructed Fermi surface pocket in an underdoped single-layer cuprate superconductor
Source: Nat Commun. 2016 Jul 22;7:12244. doi: 10.1038/ncomms12244 (PMC4961849; doi:10.1038/ncomms12244)
Supplement: Supplementary Information — Supplementary Figures 1-4, Supplementary Note 1 and Supplementary References [file ncomms12244-s1.pdf]

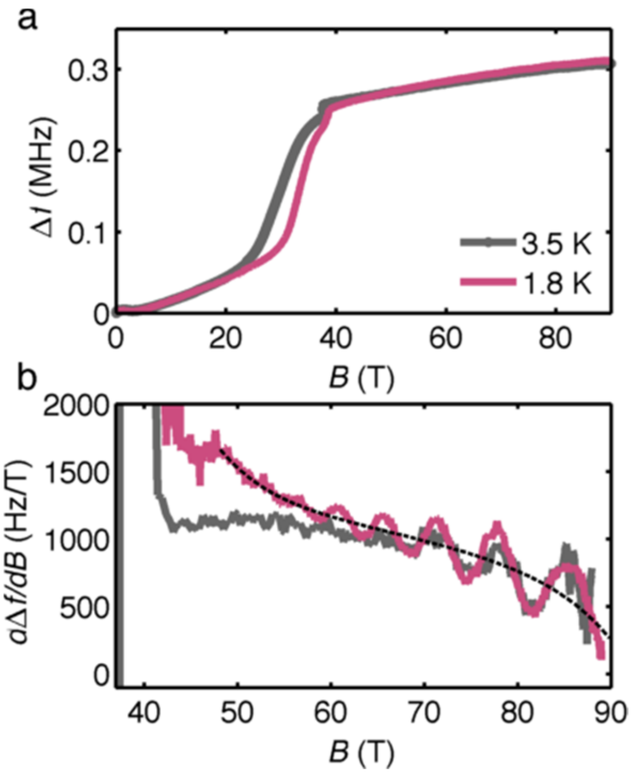

Supplemental Figure 1 | Field dependence of the non-oscillatory background for UD71. a, Raw data of the PDO circuit frequency shift as a function of applied magnetic field at 1.8 K and 3.5 K. **b.** Derivative of the raw data with respect to magnetic field reveals quantum oscillations. The derivative of the background at 1.8 K is shown as the dashed black line. The slight upturn of the 1.8 K data below ~55 T is likely due to the proximity of the superconducting transition, which creates the impression of a slowly oscillating modulation. This modulation of the background is not observed at T = 3.5 K, while the fast oscillations at high fields are still clearly visible.

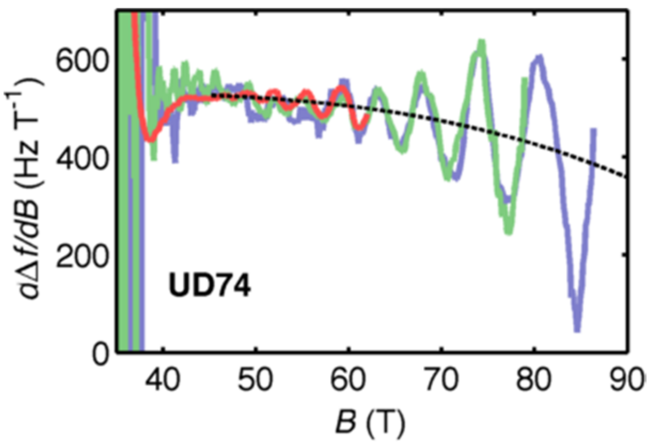

Supplemental Figure 2 | Field dependence of the non-oscillatory background for UD74. a, Derivative of the raw data for Hg1201 sample UD74. Data for three magnetic field sweeps with different maximum fields is shown. All three data sets show the same field dependence of the background (dotted black line) which does not have an oscillatory component.

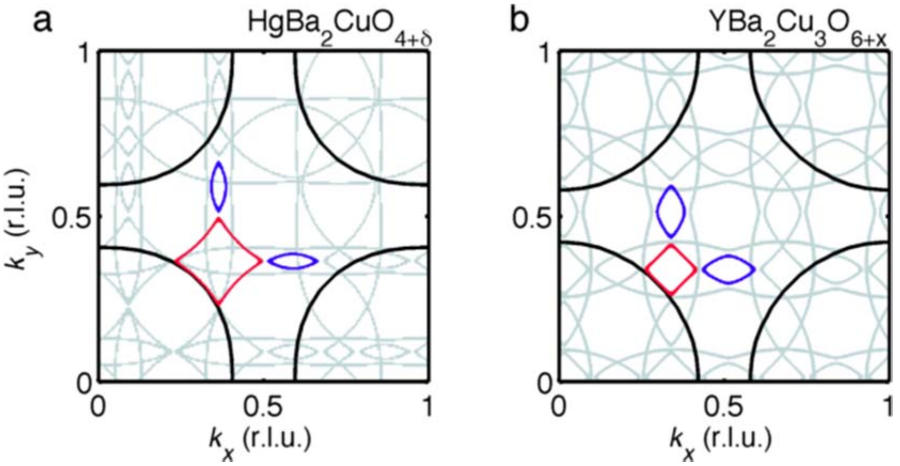

Supplemental Figure 3 | Fermi surface reconstruction by bi-axial CDW. Calculated reconstructed Fermi surface of $HgBa_2CuO_{4+\delta}$ and $YBa_2Cu_3O_{6+x}$, assuming charge ordering wave vectors (Q_{CDW} , 0) and (0 , Q_{CDW}) as discussed in the Methods section of the main text. All resulting bands are shown. We note that the additional bands that seem to overlap the electron and hole pockets in Hg1201 do not open gaps at these intersections, and thus do not break apart these pockets.

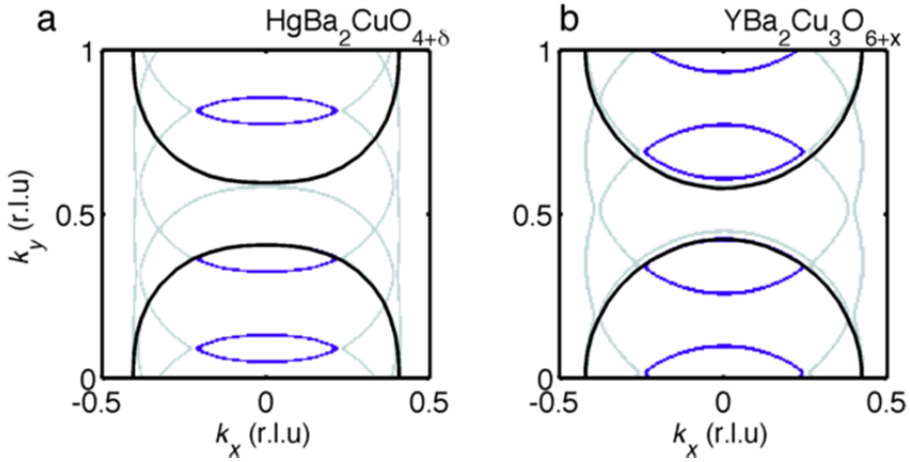

Supplemental Figure 4 | Fermi surface reconstruction by uni-axial (stripe-like) CDW. Calculated reconstructed Fermi surface of $HgBa_2CuO_{4+\delta}$ and $YBa_2Cu_3O_{6+x}$, assuming charge ordering wave vectors $(0,Q_{CDW})$. A 4x4 reconstruction is used. All resulting bands are shown. The reconstruction yields only hole-like pockets, rendered in blue.

Supplementary Note 1

Absence of small hole pockets. A recent report in Y123 assign the QO frequency $F_b \approx$ 95 T in c-axis resistivity and thermopower measurements to an additional small hole-like poket[1]. With the PDO technique on Y123, the amplitude of F_b is quite large: about 20-30% of the primary amplitude (Fig.2c and Ref.[2]). The slow modulation of our data for UD71, which we remove as a background shown as $d\Delta f/dB$ in Fig. 2b could in principle be the F_b frequency. However, the apparent increase in $d\Delta f/dB$ below 50 T at the lowest measured temperature (T = 1.8 K) is likely related to the close proximity of the superconducting transition. At a slightly elevated temperature (T = 3.5 K) the modulation of the background is no longer observed, while the fast oscillations are still clearly discernable. Since the purported small hole pocket for Y123 reported in Ref.[1] has a mass of $m^* = 0.45\ m_e$ (compared to $m^*=2.7\ m_e$ for the fast oscillations in UD71), the disappearance of the modulated background at 3.5 K in UD71 is *inconsistent* with QOs of such a light mass pocket.

Furthermore, Supplementary Fig. 2 shows $d\Delta f/dB$ for UD74 at low temperatures. It is clear that the modulation of the background is absent in UD74. The fact that the background is consistent for field sweeps to different maximum fields for UD74 rules out spurious effects such as vibrations which might obscure slow oscillations.

Supplementary References

1. N. Doiron-Leyraud *et al.*, Evidence for a small hole pocket in the fermi surface of underdoped $YBa_2Cu_3O_y$. *Nat. Commun.* 6:6034 doi: 10.1038/ncomms7034 (2015).

2. S. E. Sebastian *et al.*, Normal-state nodal electronic structure in underdoped high-T_c copper oxides. *Nature* **511**, 61-64 (2014).
